# An Easily Overlooked Contamination of Syringes in Newborn Screening by Tandem Mass Spectrometry

**DOI:** 10.3389/fped.2020.596321

**Published:** 2021-01-21

**Authors:** Yanyun Wang, Yun Sun, Tao Jiang

**Affiliations:** Nanjing Maternity and Child Health Care Hospital, Women's Hospital of Nanjing Medical University, Nanjing, China

**Keywords:** syringe, contamination, tandem mass spectrometry (MS/MS), quality control (QC), newborn screening

## Abstract

**Background:** Tandem mass spectrometry becomes a common and important test in newborn screening, but potential contamination of the equipment has largely been ignored.

**Methods:** The source of contamination through Biosan quality control samples was examined prospectively, and further confirmed by retrospective analysis of patient samples.

**Results:** We found that the source of contamination came from a syringe in the Biosan quality control samples. Furthermore, we found that a large number of indicators in the patient sample were interfered by syringe contamination in our center, and also in two other newborn screening centers, but the affected indicators were different in different screening centers.

**Conclusion:** Syringe contamination will affect the detection of patient samples by tandem mass spectrometry and should be monitored carefully and immediately.

In recent years, newborn screening has definitely become one of the most successful applications of tandem mass spectrometry (MS/MS) in the clinic (1). The measurement of amino acids (AA) and acyl carnitines (AC) by MS/MS enables the identification of over 20 inherited metabolism diseases (IMD) only a few days after birth, in one test (2). MS/MS has been under development at the Nanjing Newborn Screening Center since 2013, including two sets of MS/MS TQD and Xevo-TQD (XTQD).

## MS/MS Detection Platform in Jiangsu Province and Zhejiang Province

**Figure 1:**
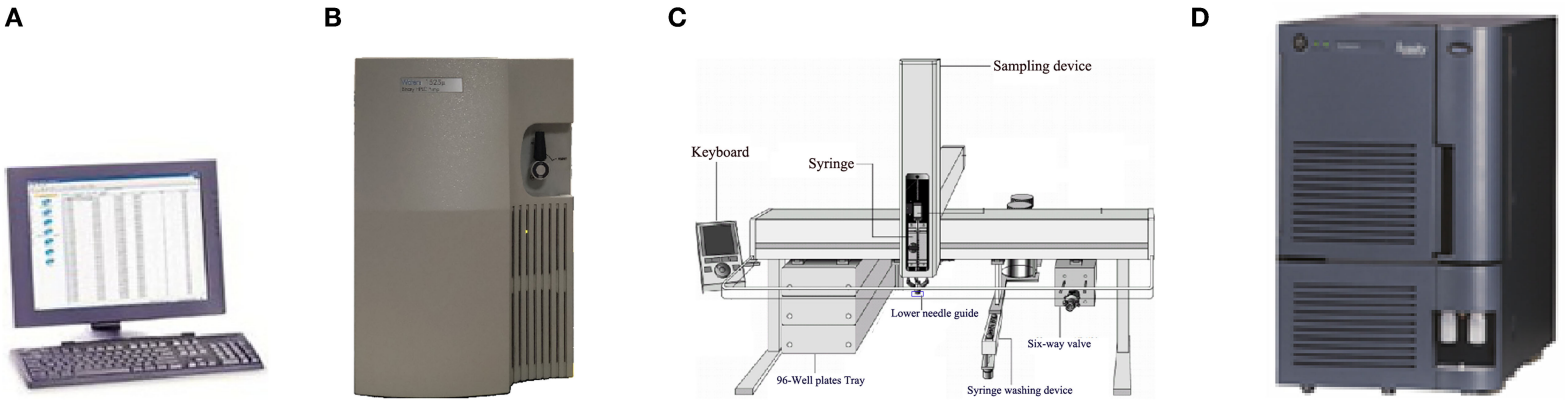
Exploded view of MS/MS detection platform [**(A)**: Computer provided by Waters corporation; **(B)**: 1,525 μ high performance liquid pump of Waters corporation; **(C)**: 2,777C samples manager; **(D)**: Mass spectrometer of Waters Corporation].

The Nanjing Newborn Screening Center is one of 13 screening centers in Jiangsu Province. Newborn screening centers in Jiangsu Province and Zhejiang Province both use the Tandem Mass Spectrometry (MS/MS) platform of Waters Corporation. The platform consists of four parts: instrument control and a data processing system (computer), a 1,525 μ high performance liquid pump, 2,777C samples manager and tandem mass spectrometry (Figure 1).

Generally speaking, the 2,777C samples manager has three components: injection needle, a valve, and the peek tube. One syringe (injection needle) in the samples manager is used to complete the injection of the samples. The main body of the syringe is made of glass and stainless steel which are anticorrosion materials, and means therefore, that the syringe will not be replaced until it is damaged (Supplementary Figure 1). The injection needle is a consumable material in the sample management and is made up of two components: a glass barrel and a plunger. The plunger has to be regularly replaced, without needing to replace the glass barrel. Therefore, the glass barrel could be contaminated with the samples, but is not noticed or reported.

A range of different interferences in newborn screening has been reported. For example, the impact of punch location (3), sample volume (4), EDTA in dry blood spots (DBS) (5), and contamination of the DBS (6). However, contamination of the syringe has never been reported. The purpose of this study was to determine whether syringe contamination interferes with MS/MS detection and to establish a model for regular maintenance of syringes.

## The Process of Eliminating Possible Contamination

Samples were taken from 96-well plates by a syringe, mixed with flow solvent in the valves, delivered to mass spectrometry via the peek tube, and analyzed by mass spectrometry ionizing through the ESI-SS capillary (Supplementary Figure 2). Accordingly, the possible contaminated parts before MS/MS analysis were as follows: syringe, lower needle guide, valves, peek tube, and ESI-SS capillary.

## Methods

**Figure 2:**
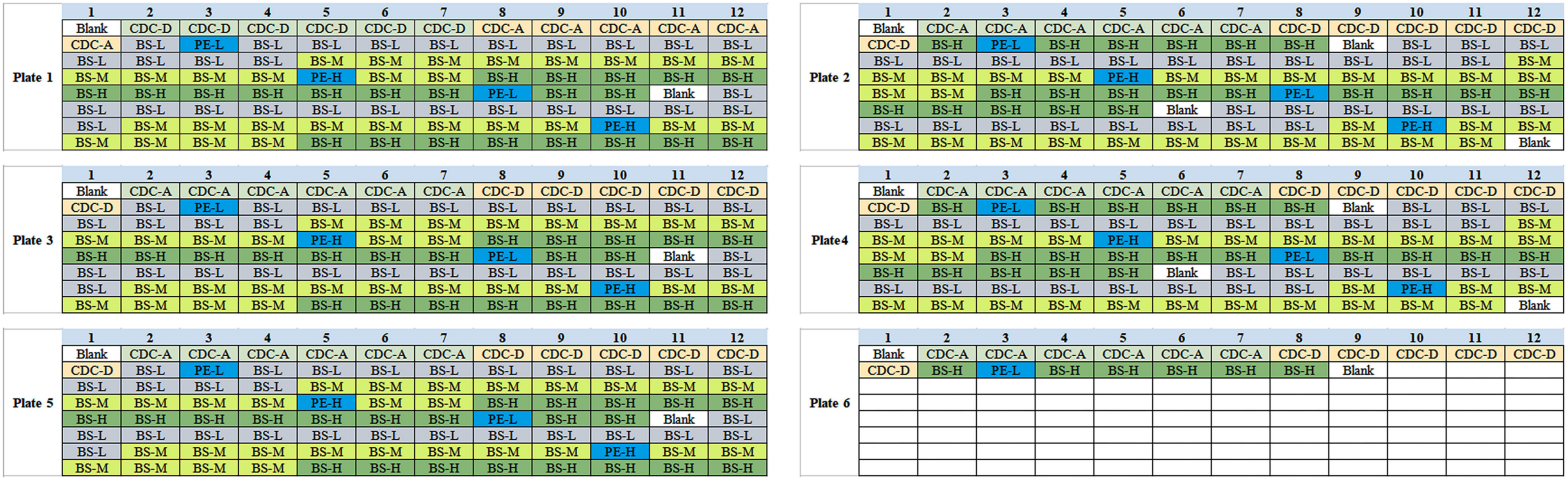
Schematic diagram of Biosan QC samples in 96-well plates.

**Figure 3:**
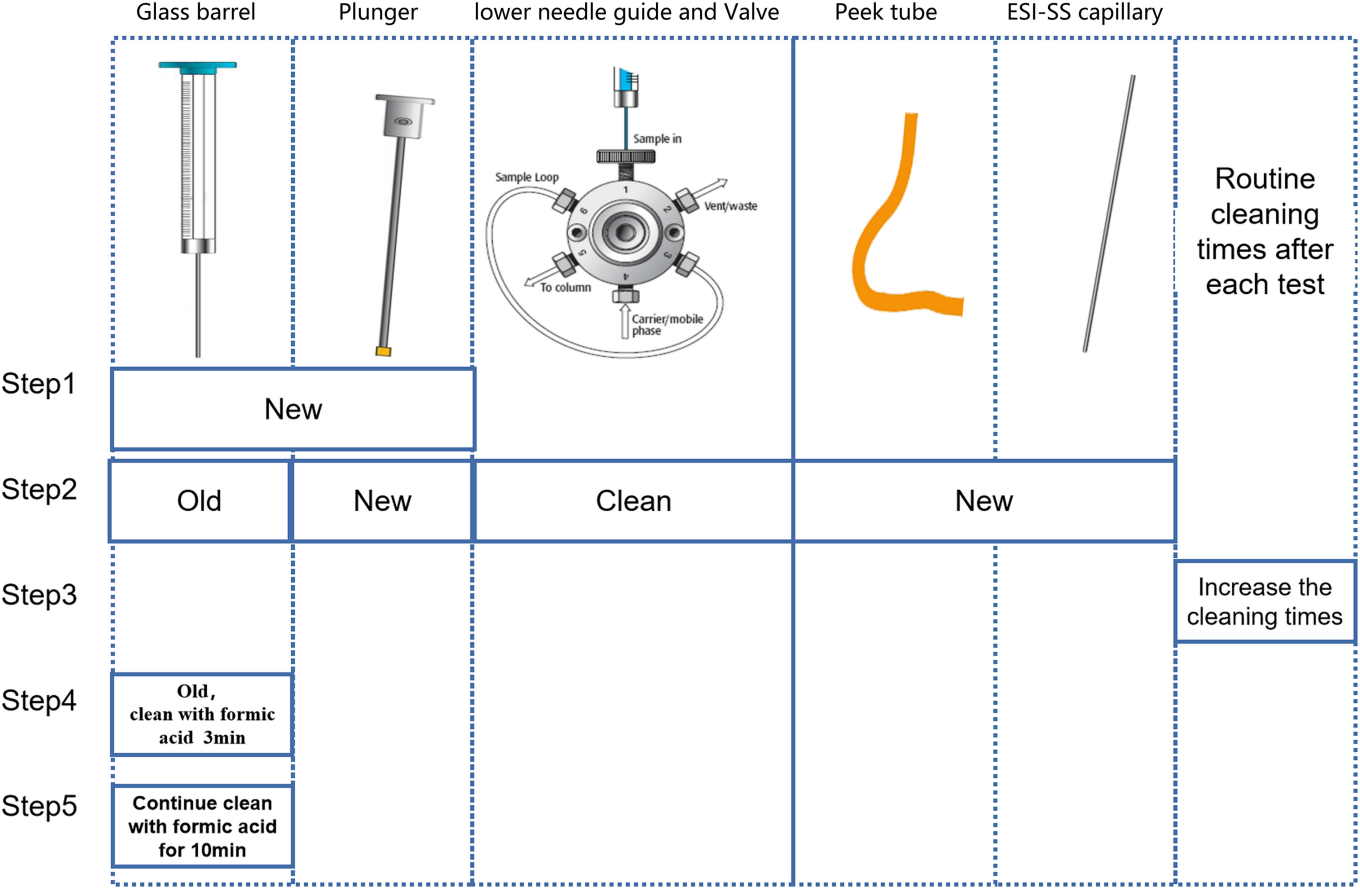
Schematic diagram of troubleshooting steps.

1. Amino acids and acyl carnitines were detected by commercial non-derivatized MS/MS kits (PerkinElmer, PE company) as described previously (7). Quality control (QC) relied on measurements of PE and American CDC control samples. The third internal QC sample was provided by the Biosan company which was divided into three concentration groups as low (L), middle (M) and high (H). Detection indexes in Biosan's samples contained 11 kinds of amino acids (Ala, Arg, Cit, Leu, Met, Orn, Phe, Tyr, Val, Gly, and Pro), 16 kinds of acyl carnitine (C0, C2, C3, C4, C5, C5DC+C6OH, C4DC+C5OH, C6, C8, C10, C12, C14, C16, C18) and succinylacetone (SA). Each set of the Biosan sample contains two “big” dry blood spots of L, M, and H concentration. The diameter of each “big” blood spot is not < 13.0 mm. In testing, a “small” blood spot with a diameter of 3.2 mm needs to be cut from the “big” blood spot as a test sample by P9 (panthera-puncherTM 9TM, PE company). The samples were processed by P9 as shown in Supplementary Figure 3. Ten sets of Biosan samples were taken randomly with the same batch number, and the layout of a total of 140 blood spots are shown in Figure 2. Each 96-well plate included internal QC samples from the American CDC and PE to ensure the accuracy and comparability of the data. Samples of Biosan were distributed in the order of 14 low QC (BS-L) - 14 middle QC (BS-M) - 14 high QC (BS-H).

2. There are five parts that need to be checked step by step. The troubleshooting was established step by step following 2.1–2.5 (Figure 3). For each step, we arranged two blank samples and two Biosan low QC samples with a PE high QC sample (Table 1).

**Table 1:** Schematic diagram of samples arrangement position in troubleshooting experiments.

	**1**	**2**	**3**	**4**	**5**	**6**	**7**	**8**	**9**	**10**	**11**	**12**
A	Blank 1	Blank 2	Blank 3	PE-H 1	Blank 4	Blank 5	PE-H 2	Blank 6	Blank 7	PE-H 3	BS-L 1	BS-L 2
	PE-H 4	BS-L 3	BS-L 4									
B	PE-H 5	Blank 8	Blank 9	PE-H 6	Blank 10	Blank 11	PE-H 7	BS-L 5	BS-L 6	PE-H 8	BS-L 7	BS-L 8
C	PE-H 9	Blank 12	Blank 13	PE-H 10	Blank 14	Blank 15	PE-H 11	BS-L 9	BS-L 10	PE-H 12	BS-L 11	BS-L 12
D	PE-H 13	Blank 16	Blank 17	PE-H 14	Blank 18	Blank 19	PE-H 15	BS-L 13	BS-L 14	PE-H 16	BS-L 15	BS-L 16
E	PE-H 17	Blank 20	Blank 21	PE-H 18	Blank 22	Blank 23	PE-H 19	BS-L 17	BS-L 18	PE-H 20	BS-L 19	BS-L 20
F												
G												

*PE-H, PE-high-QC sample; BS-L, BS-low-QC samples*.

2.1 Just replacing the old syringe with a new syringe.

2.2 Using the old syringe, cleaning the lower needle guide and valves ultrasonically several times, and changing the plunger, peek tube, and ESI-SS capillary.

2.3 Using the old syringe and washing it two to four times after the injection of the PE high QC sample.

2.4 Using the old syringe and cleaning the old syringe with anhydrous formic acid (Aladdin, 64-18-6) for 3 min before drawing samples.

2.5 Using the old syringe and cleaning the old syringe with anhydrous formic acid for 10 min before drawing samples.

3. This study was approved by the Ethical committee of Nanjing Maternity and Child Health Care Hospital, and 50,000 consecutive cases of newborn screening in 2018 were recruited.

## Results

**Figure 4:**
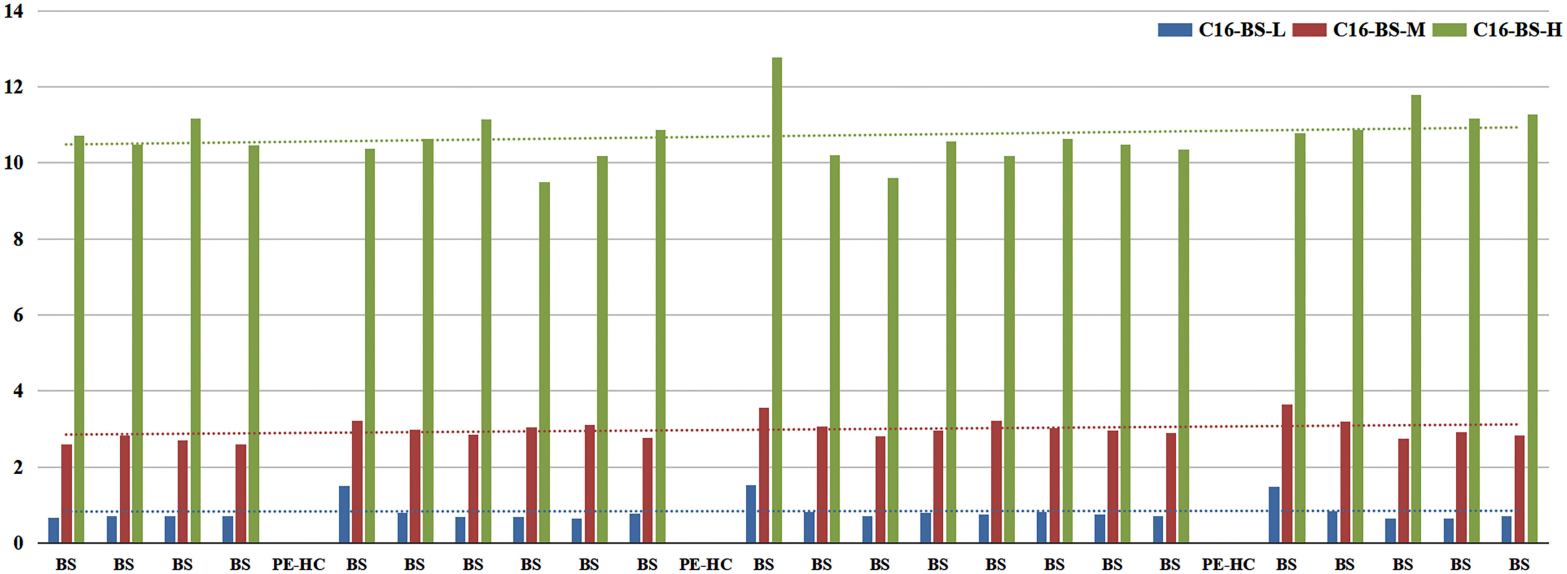
Column chart of C16 value detected before and after the PE-high-QC samples.

1. Basic values of each index are shown in Table 2. In all indicators, the coefficient of variation (CV) in C16 was the highest. Furthermore, CV of BS-middle-QC and BS-low-QC samples was higher than BS-high-QC. The data were sorted in the original order in 96-well plates. It is worth noting that C16 in the first sample after the PE-high-QC sample was in particular higher than others, especially in BS-low-QC samples (Figure 4). This was the reason the CV in C16 was so high.

**Table 2:** Basic values of each index.

**Index**	**L-X**	**L-SD**	**L-CV (%)**	**M-X**	**M-SD**	**M-CV**	**H-X**	**H-SD**	**H-CV**
ALA	436.68	24.8	5.68	1,099.76	63.23	5.75%	1,554.76	87.20	5.61%
ARG	8.36	0.54	6.44	67.04	3.47	5.17%	174.57	9.42	5.39%
CIT	38.31	2.15	5.62	137.33	7.35	5.35%	727.30	40.70	5.60%
LEU	202.62	10.57	5.22	372.54	19.93	5.35%	994.36	59.81	6.02%
MET	15.85	0.96	6.08	43.63	2.46	5.64%	264.45	13.59	5.14%
ORN	144.57	8.33	5.76	444.58	24.06	5.41%	1,594.27	102.32	6.42%
PHE	56.72	3	5.3	105.85	5.57	5.27%	536.74	28.74	5.36%
TYR	141.54	7.13	5.04	325.28	17.66	5.43%	825.28	46.51	5.64%
VAL	204.11	10.71	5.25	356.77	19.61	5.50%	786.00	48.84	6.21%
GLY	299.57	17.99	6	1,014.02	59.51	5.87%	1,586.02	95.94	6.05%
PRO	134.42	7.29	5.42	540.44	29.12	5.39%	1,444.08	78.61	5.44%
C0	13.37	0.74	5.55	57.44	3.10	5.39%	126.33	6.36	5.04%
C2	41	2.17	5.29	74.21	4.09	5.52%	152.02	8.87	5.83%
C3	1.97	0.12	6.17	6.21	0.37	5.88%	10.91	0.63	5.80%
C4	0.38	0.03	7.17	1.00	0.06	6.02%	3.41	0.21	6.29%
C5	0.32	0.02	6.7	1.06	0.05	4.79%	5.88	0.30	5.17%
C5DC+C6OH	0.24	0.02	9.06	0.88	0.06	6.58%	4.09	0.23	5.75%
C4DC+C5OH	0.32	0.01	6.25	0.95	0.04	4.54%	4.96	0.25	4.98%
C6	0.14	0.01	9.06	0.42	0.03	7.35%	1.02	0.06	5.61%
C8	0.54	0.03	6.18	1.39	0.08	5.94%	2.21	0.14	6.37%
C10	0.33	0.02	7.08	0.86	0.05	6.30%	1.39	0.08	6.10%
C12	0.26	0.02	6.36	0.67	0.04	6.25%	1.14	0.08	6.72%
C14	0.52	0.04	7.61	1.72	0.13	7.54%	4.60	0.33	7.28%
C16	0.73	0.07	**9.72**	2.96	0.28	**9.45%**	10.78	0.76	7.09%
C16OH	0.14	0.01	7.9	0.66	0.04	6.30%	2.18	0.15	7.01%
C18	0.61	0.05	8.95	1.41	0.10	7.23%	3.69	0.28	7.49%
SA	1.1	0.09	8.35	2.56	0.18	7.05%	9.19	0.58	6.33%

*L-X, Mean of BS-low-QC samples; L-SD, Standard Deviation of BS-low-QC samples; L-CV, Coefficient of Variation of BS-low-QC samples; M-X, Mean of BS-medium-QC samples; M-SD, Standard Deviation of BS-medium-QC samples; M-CV, Coefficient of Variation of BS-medium-QC samples; H-X, Mean of BS-high-QC samples; H-SD, Standard Deviation of BS-high-QC samples; H-CV, Coefficient of Variation of BS-high-QC samples. C16 had the highest L-CV and M-CV values which were marked in bold*.

**Figure 5:**
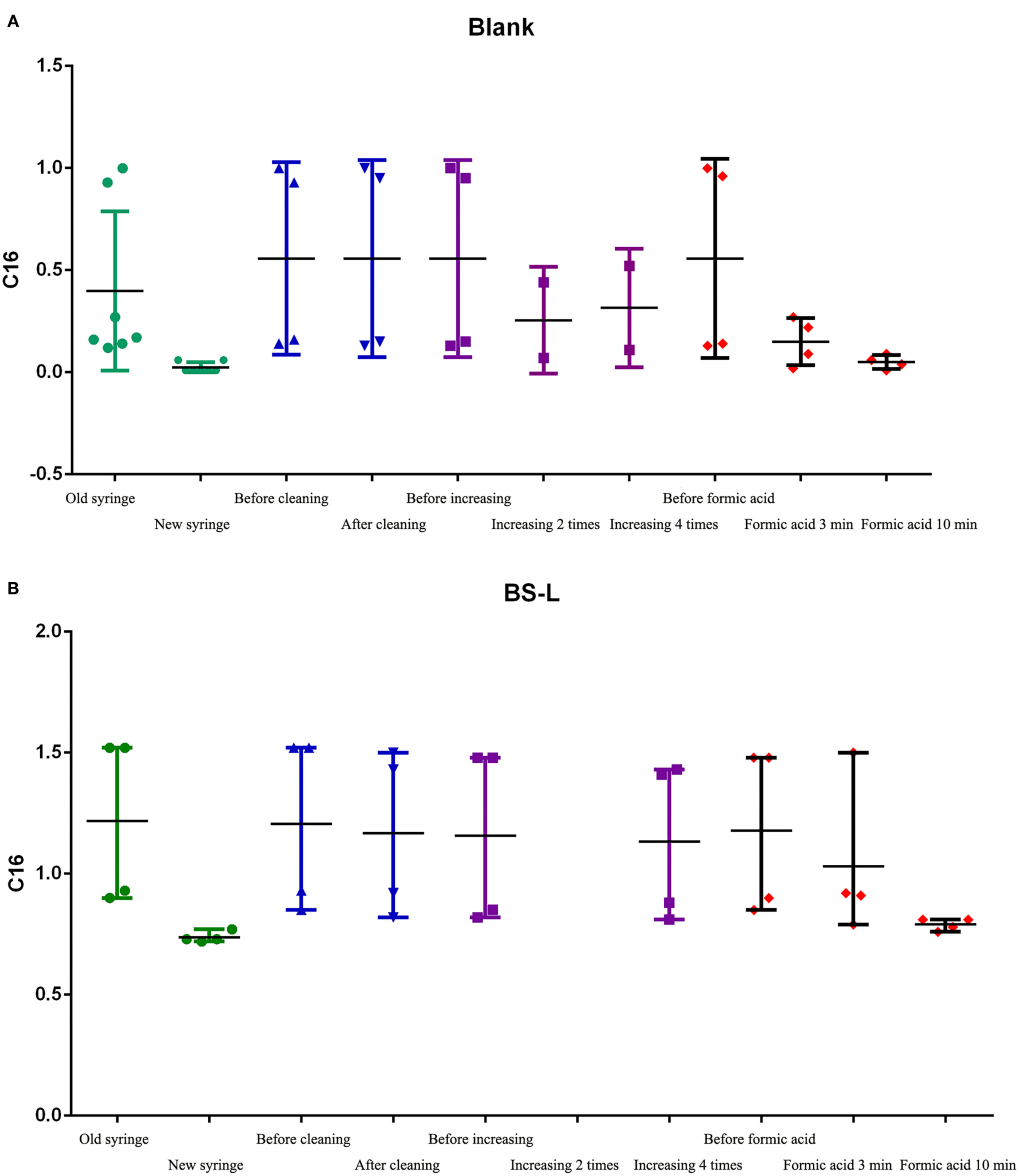
The C16 results of steps 2.1-2.5. **(A)** The results of C16 in Blank samples. **(B)** The results of C16 in BS-L-QC samples.

2. Therefore, in subsequent steps, to identify the source of contamination, we mainly analyzed C16 in BS-low-QC samples. The results of steps 2.1–2.5 are shown in Figure 5.

2.1 Test results of replacing the syringe: after syringe replacement, the detection of C16 in Blank-samples and BS-L samples was significantly lower than before syringe replacement and was close to the mean of C16 in Table 2 (0.73).

2.2 Test results of C16 with the old syringe, after cleaning the lower needle guide and valves, changing the plunger, peek tube, and ESI-SS capillary: Before and after cleaning, C16 in Blank-samples and BS-L samples had no obvious difference, suggesting that the above parts were not contaminated.

2.3 Test results of C16 with the old syringe, after increasing washing to between two and four times: The value of C16 had changed slightly, but it failed to reach normal levels as shown in Table 2. The results suggest that the number of times washed is not the primary factor in contamination.

2.4 and 2.5 Test results of C16 with the old syringe, after cleaning the old syringe by formic acid before drawing samples: After repeated cleaning by formic acid, the color of formic acid solution changed from colorless to pink (Supplementary Figure 4). The value of C16 changed significantly after cleaning by formic acid for 3 min but returned to normal after cleaning by formic acid for 10 min. These results suggest that the syringe is the primary factor of carrying contamination, especially the glass barrel.

It is also worth noting that the value of the second sample after the PE-high-QC sample was close to the normal value.

3. Finally, 53,111 cases were included in the retrospective analysis. The concentrations of the first sample after PE-high-QC were significantly higher than other samples, especially C8:1, C10, C12, C12:1, C14, C14:1, C16, C18, and C18:1OH with a bias over 10% (Table 3).

**Table 3:** The concentration of the first sample after PE-high-QC and other samples.

**Index**	**Median**				
	**A: Total (n = 53,111)**	**B: The first sample after PE-high-QC 样本 (*n* = 1,146)**	**C: Other sample (*n* = 51,965)**	**Multiple**	**Bias (B-C)/C%**
ALA	316.63	320.86	316.52	1.01	1.37%
ARG	9.05	8.96	9.06	0.99	−1.10%
CIT	12.94	13.63	12.92	1.05	5.46%
GLY	470.8	481.05	470.52	1.02	2.24%
LEU+ILE+PRO-OH	134.37	134.99	134.35	1	0.47%
MET	20.55	21.68	20.52	1.06	5.63%
ORN	104.07	104.7	104.07	1.01	0.60%
PHE	48.06	49.84	48.03	1.04	3.76%
PRO	193.36	197.81	193.26	1.02	2.35%
SA	0.77	0.81	0.77	1.05	5.19%
TYR	93.72	95.73	93.68	1.02	2.18%
VAL	126.15	127.6	126.1	1.01	1.19%
C0	19.83	20.27	19.82	1.02	2.25%
C2	18.32	18.76	18.31	1.02	2.46%
C3	1.51	1.59	1.51	1.05	4.97%
C3DC+C4OH	0.1	0.1	0.1	1	0.00%
C4	0.2	0.21	0.2	1.05	5.00%
C4DC+C5OH	0.2	0.2	0.2	1	0.00%
C5	0.1	0.1	0.1	1	0.00%
C5:1	0.01	0.01	0.01	1	0.00%
C5DC+C6OH	0.1	0.1	0.1	1	0.00%
C6	0.04	0.04	0.04	1	0.00%
C6DC	0.09	0.09	0.09	1	0.00%
C8	0.05	0.05	0.05	1	0.00%
C8:1	0.09	0.1	0.09	1.11	**11.11%**
C10	0.07	0.08	0.07	1.14	**14.29%**
C10:1	0.06	0.06	0.06	1	0.00%
C10:2	0.01	0.01	0.01	1	0.00%
C12	0.07	0.09	0.07	1.29	**28.57%**
C12:1	0.04	0.05	0.04	1.25	**25.00%**
C14	0.17	0.22	0.17	1.29	**29.41%**
C14:1	0.08	0.09	0.08	1.13	**12.50%**
C14:2	0.02	0.02	0.02	1	0.00%
C14OH	0.01	0.01	0.01	1	0.00%
C16	2.87	3.67	2.85	1.29	**28.77%**
C16:1	0.16	0.16	0.16	1	0.00%
C16:1OH	0.03	0.03	0.03	1	0.00%
C16OH	0.02	0.02	0.02	1	0.00%
C18	0.82	1.02	0.82	1.24	**24.39%**
C18:1	1.34	1.33	1.34	0.99	−0.75%
C18:1OH	0.02	0.04	0.02	2	**100.00%**
C18:2	0.2	0.19	0.2	0.95	−5.00%
C18OH	0.01	0.01	0.01	1	0.00%

*(B−C)/C values over 10% were marked in bold*.

In order to confirm our findings, we contacted two other Newborn Screening Centers. We found similar results, but the indexes with a bias over 10% were different. CIT, C4, C5, C5DC+C6OH, C8, C10, C12, C14, C14:1, and C18:1OH were found in one Newborn Screening Centre, but ARG, CIT, SA, TYR, C2, C3DC+C4OH, C4, C4DC+C5OH, C5DC+C6OH, C6DC, C12, C14, C16, C16:1, C16:1OH, C18, C18:1, C18:1OH, and C18:2 were found in the other Newborn Screening Centre.

4 Applying the research results to the clinic.

**Figure 6:**
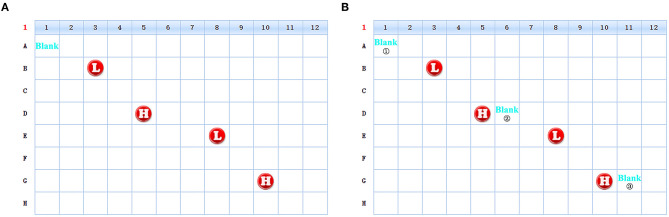
The schematic diagram of the change of the sample position in 96-well plate. [**(A)** Schematic diagram of old version scheme. **(B)** Schematic diagram of new version scheme. The unmarked locations in the figures are all screening samples].

4.1 All indicators in the PE-high-QC are several times higher than the screening sample. The first well sample after the PE-high-QC might be contaminated by the syringe. In order to avoid this interference, the Blank well is set between PE-high-QC and the screening sample (Figure 6).

4.2 Appling the research results to Routine maintenance. After each experiment, Blank ①, ②, and ③ in Figure 6 were compared in order to monitor the changes. When Blank ② or ③ was significantly higher than Blank ①, the Syringe was cleaned with formic acid and the cleaning time was 10 min each time (Supplementary Figure 5). The Syringe is replaced once a year.

## Discussion

In this study, the contamination of syringes was identified by BS-QC samples and patient data. The syringe is generally cleaned with 50% methanol-water mixtures in ordinary maintenance. However, as the sample size increases, the sample residue would remain in the internal part of the glass barrel, especially in the fixation of the needle to the barrel. In addition, as the plunger ages, the tightness of the syringe is reduced, which could exacerbate the sample residue, especially after injecting the PE-high-QC. Therefore, it is necessary to pay attention to the cleaning, maintenance, and replacement of syringe in clinical practice.

Based on the differences in concentrations of patient samples and PE-QC, especially PE-high-QC, we found that the carrying effect of PE-high-QC mainly affected the first patient sample while the subsequent samples were not affected. Therefore, we suggest adding one blank sample after the PE-high-QC. The mode of detection could change from “PE-high-QC**→**patient sample” to “PE-high-QC**→**Blank**→**patient sample.” In addition, we should examine the fluctuation of indexes in the Blank sample, such as C16. If the concentration of some indexes in the Blank sample increase significantly after the PE-high-QC, we should clean the syringe with formic acid immediately.

Since the fore-end of the plunger is a rubber plug, it wears out and the tightness of syringe reduces after long-term use. Therefore, we should pay attention to the wear condition of plungers and replace it in time. We recommend replacing the glass barrel every 1–2 years.

## Consent for Publication

The study did not involve human participants. The research subject is focused around the quality control of materials which came from the United States CDC, Pthe ekinElmer company, and the Biosan company.

## Data Availability Statement

The original contributions presented in the study are included in the article/Supplementary Material, further inquiries can be directed to the corresponding author/s.

## Ethics Statement

This study was approved by Ethical committee of Nanjing Maternity and Child Health Care Hospital, and 50,000 consecutive cases of newborn screening in 2018 were recruited.

## Author Contributions

YW led the review process, drafted the initial manuscript, and extracted data. YS reviewed the draft. TJ designed the study. All authors read and approved the final manuscript.

## Conflict of Interest

The authors declare that the research was conducted in the absence of any commercial or financial relationships that could be construed as a potential conflict of interest.

## Supplementary Material

Supplementary Figure 1 Schematic diagram of the syringe which is composed of glass barrel and plunger.

Supplementary Figure 2 The possible contamination parts before MS/MS analysis.

Supplementary Figure 3 Schematic diagram of punch position in dry blood spot of Biosan QC sample (6 “small” blood spot samples were drilled for each “big” blood spot to avoid the center position).

Supplementary Figure 4 Color change of solution before and after cleaning by formic acid. **(A)** Manual cleaning. The formic acid before cleaning was colorless and transparent. **(B)** The solution after cleaning turned pink.

Supplementary Figure 5 Schematic diagram of changes of ALA and C16 in Blank before and after cleaning with formic acid.
