# Supplementary figures and images for: An Easily Overlooked Contamination of Syringes in Newborn Screening by Tandem Mass Spectrometry
Source: Front Pediatr. 2021 Jan 21;8:596321. doi: 10.3389/fped.2020.596321 (PMC7861201; doi:10.3389/fped.2020.596321)

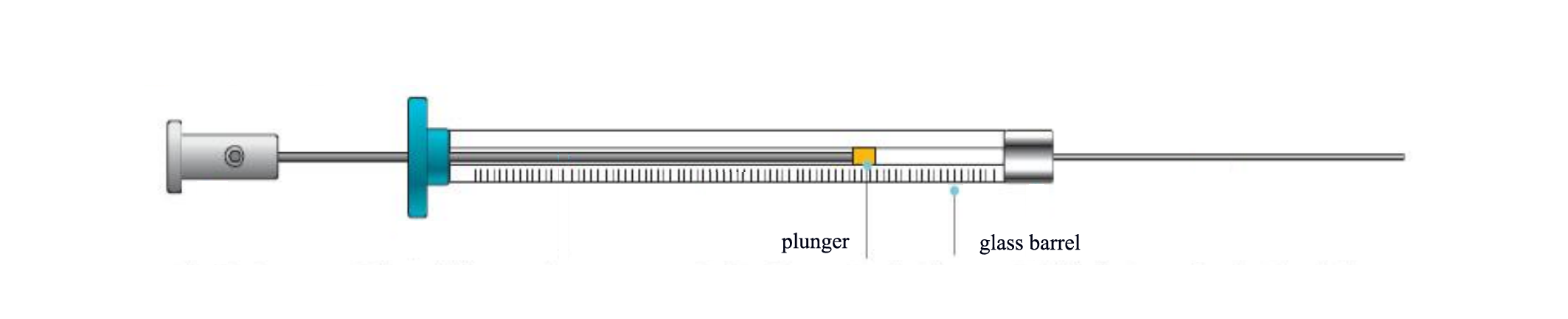

Supplement: Supplementary Figure 1 — Schematic diagram of the syringe which is composed of glass barrel and plunger. [file Image_1.TIF]

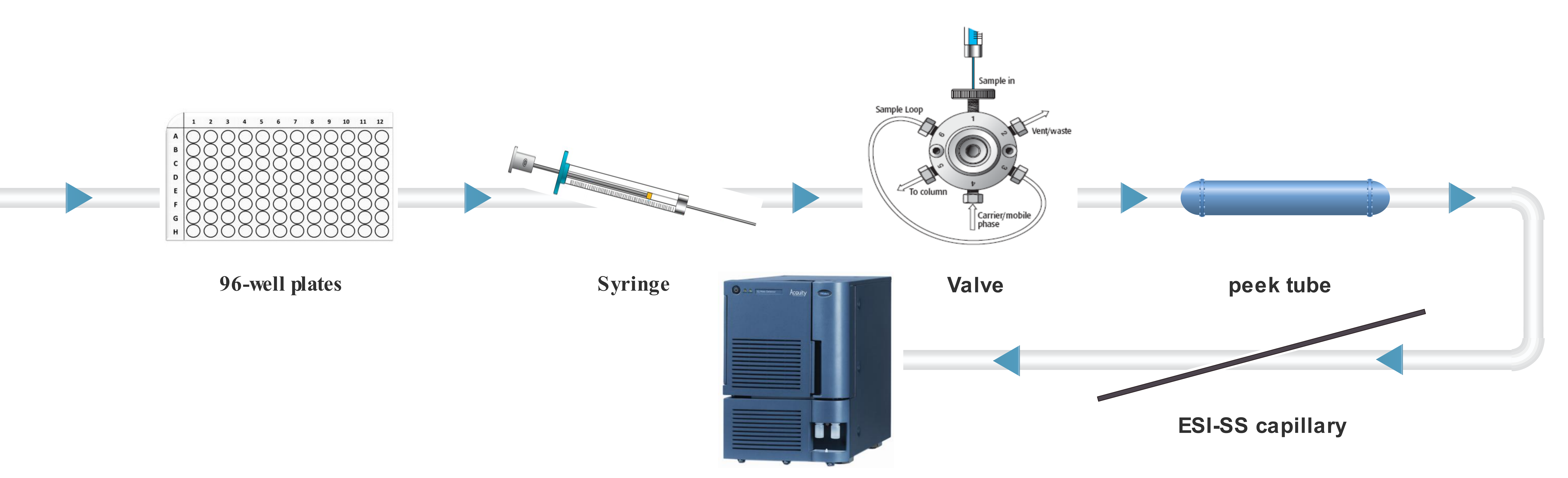

Supplement: Supplementary Figure 2 — The possible contamination parts before MS/MS analysis. [file Image_2.png]

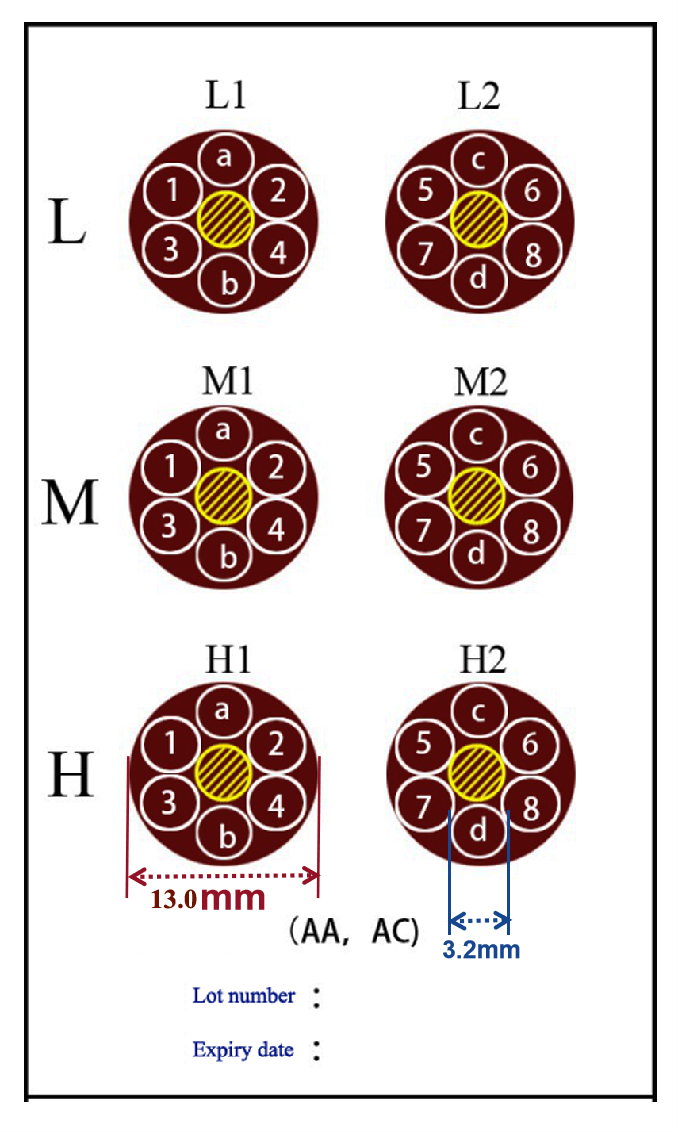

Supplement: Supplementary Figure 3 — Schematic diagram of punch position in dry blood spot of Biosan QC sample (6 “small” blood spot samples were drilled for each “big” blood spot to avoid the center position). [file Image_3.TIF]

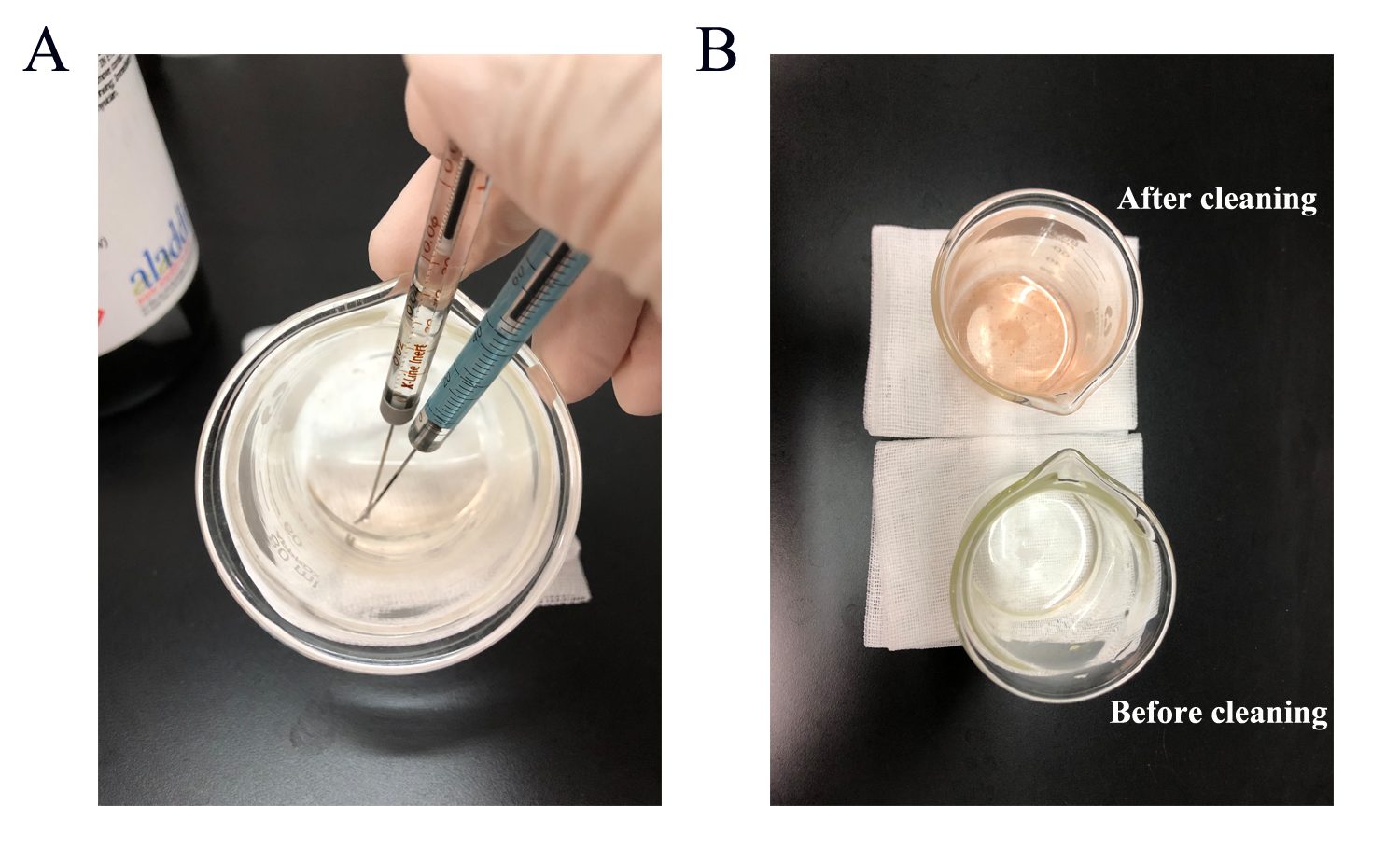

Supplement: Supplementary Figure 4 — Color change of solution before and after cleaning by formic acid. (A) Manual cleaning. The formic acid before cleaning was colorless and transparent. (B) The solution after cleaning turned pink. [file Image_4.TIF]

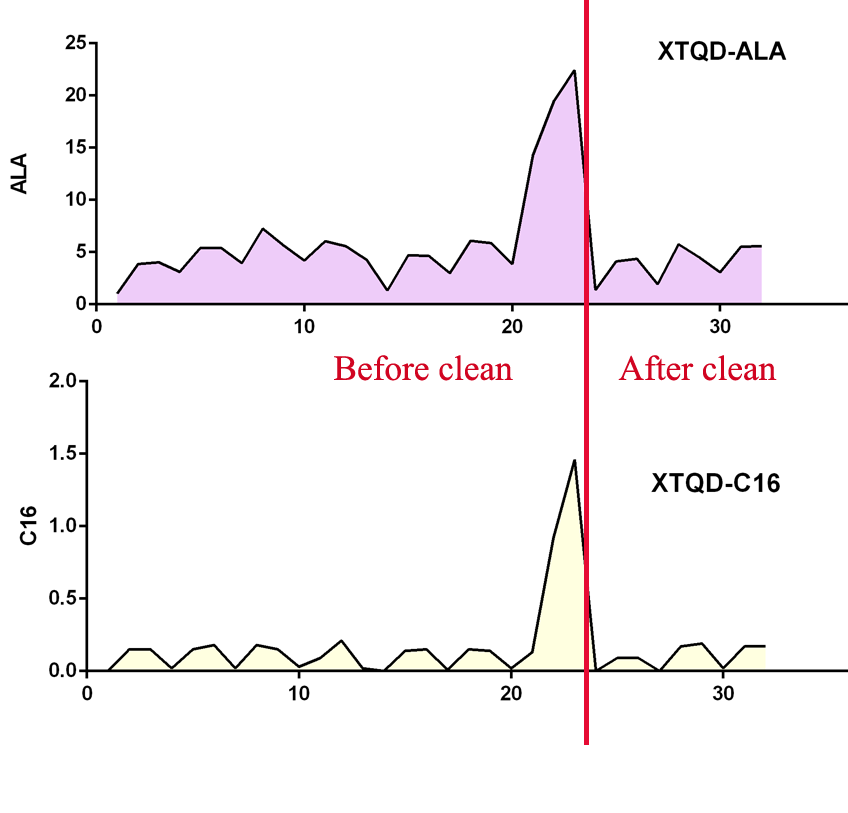

Supplement: Supplementary Figure 5 — Schematic diagram of changes of ALA and C16 in Blank before and after cleaning with formic acid. [file Image_5.TIF]
